# Supplementary material for: An informatics consult approach for generating clinical evidence for treatment decisions
Source: BMC Med Inform Decis Mak. 2021 Oct 12;21:281. doi: 10.1186/s12911-021-01638-z (PMC8506488; doi:10.1186/s12911-021-01638-z)
Supplement: Supplementary file 1 — Additional file 1. Supplementary data 1 to 11. [file 12911_2021_1638_MOESM1_ESM.pdf]

Additional file 1: Comparison of the Informatics Consult with traditional approaches to evidence generation.

Areas	Traditional methods of evidence generation	Informatics Consult
**Who initiates the question?**	Top down: typically driven by a small number of researchers	Bottom up: democratic, any clinician, any uncertainty and potentially patient driven
**Simultaneous delivery of all relevant evidence**	Not often possible, one study and one design at a time	Yes
**Embedded in health systems (the question or the answer or the means of providing answer)**	No	Yes
**Clinical trial prioritisation, informing trial design and recruitment**	Top down: driven by specific groups of individuals	Bottom up: opportunities for embedding within clinical practice
**Involvement of individuals**	Researcher centric	Patient and clinician centric
**Strength of evidence and levels of evidence**	Levels of evidence classified in all major clinical guidelines; however, these are all based on peer reviewed published evidence	Same levels of evidence apply; however, the framework aims to return evidence within clinical timescales to mitigate hurdles in the journal peer-review process. Potential for collating information from past Consults in an open-access repository.
**Clinical guideline recommendations based on evidence**	Yes, information from randomised trials and peer-reviewed publications are included in clinical guidelines	Raises important questions on what basis might clinicians follow the Consult before it is peer reviewed and published
**Regulatory approvals on the labels of drugs and medical devices**	Food and Drug Administration (FDA), European Medicines Agency and Medicines and Healthcare products Regulatory Agency established processes for trials and for the Real-world Evidence framework	Largely untested

Additional file 2: Specification and emulation of a target trial of warfarin therapy and mortality or stroke risk using

Protocol component	Target trial specificiation	Target trial emulation
**Eligibility criteria**	Age ≥ 30 between 1 January 1998 and 27 June 2016	Same as for the target trial
	Age ≤ 85 at baseline	
	No history of stroke at baseline	
	At least 1 year of up-to-standard data in a CPRD practice	
	At least 1 year of potential follow-up from baseline	
	Baseline is defined as the latest date by which an individual is diagnosed with cirrhosis or atrial fibrillation + 3 months (individuals must have both conditions at baseline), given that all eligibility criteria are met.	
**Treatment strategies**	Initiation of warfarin therapy versus no initiation of warfarin at baseline	Same as for the target trial. Date of medication initiation is defined as the first date of a prescription.
**Treatment assignment**	Individuals are randomly assigned to a strategy at baseline	Randomisation is emulated by adjusting for baseline confounders.
**Outcomes**	All-cause mortality and incident ischaemic stroke.	Same as for the target trial
**Follow-up**	Starts at baseline and ends at the time of reaching an outcome, death, loss to follow-up (transfer out of the practice) or administrative end of follow-up (end of practice data collection or 27 June 2016), whichever happens first.	Same as for the target trial
**Causal contrast**	Intention-to-treat effect	Observational analog for the intention-to-treat effect
**Statistical analyses**	Intention-to-treat analysis	Same as for the target trial, adjusting for baseline covariates via propensity score or regression adjustment.
	Subgroup analyses by age (< 65 or ≥ 65), sex, normal INR (< 1.7).	Same subgroup analyses.

Additional file 3: Baseline characteristics of the unmatched and matched cohorts

	Unmatched cohort			Matched cohort		
**Characteristics**	**No warfarin**	**Warfarin**	**Standardised difference**	**No warfarin**	**Warfarin**	**Standardised difference**
**N**	579	443		526	235	
**Gender, women (%)**	199 (34.4)	180 (40.6)	0.13	193 (36.7)	95 (40.4)	0.077
**Age at baseline, years (mean (SD))**	65.66 (12.50)	61.69 (13.42)	0.306	65.16 (12.56)	61.11 (13.89)	0.306
**Type 2 diabetes (%)**	136 (23.5)	99 (22.3)	0.027	125 (23.8)	46 (19.6)	0.102
**Hypertension (%)**	258 (44.6)	183 (41.3)	0.066	220 (41.8)	80 (34.0)	0.161
**Ascites (%)**	143 (24.7)	125 (28.2)	0.08	130 (24.7)	67 (28.5)	0.086
**Hepatic encephalopathy (%)**	29 (5.0)	32 (7.2)	0.093	26 (4.9)	14 (6.0)	0.045
**Renal disease (%)**	78 (13.5)	58 (13.1)	0.011	62 (11.8)	21 (8.9)	0.094
**Steatosis (%)**	63 (10.9)	48 (10.8)	0.001	56 (10.6)	27 (11.5)	0.027
**Lipid regulating drugs (%)**	160 (27.6)	130 (29.3)	0.038	144 (27.4)	58 (24.7)	0.061
**Hypertension treatment (%)**	236 (40.8)	194 (43.8)	0.061	216 (41.1)	94 (40.0)	0.022
**Digoxin (%)**	107 (18.5)	65 (14.7)	0.103	95 (18.1)	27 (11.5)	0.186
**Varices (%)**	95 (16.4)	74 (16.7)	0.008	81 (15.4)	33 (14.0)	0.038
**Bilirubin ≥ 34.2 μmol/L (%)**	130 (22.5)	93 (21.0)	0.035	110 (20.9)	42 (17.9)	0.077
**Albumin < 35 g/L (%)**	18 (3.1)	21 (4.7)	0.084	17 (3.2)	12 (5.1)	0.094
**INR ≥ 1.7 (%)**	11 (1.9)	217 (49.0)	1.285	11 (2.1)	12 (5.1)	0.162
**Alanine aminotransferase ≥ 35 U/L (%)**	246 (42.5)	179 (40.4)	0.042	211 (40.1)	84 (35.7)	0.09
**Aspartate transaminase ≥ 40 U/L (%)**	119 (20.6)	88 (19.9)	0.017	104 (19.8)	38 (16.2)	0.094
**BMI ≥ 30 kg/m2 (%)**	227 (39.2)	169 (38.1)	0.022	208 (39.5)	81 (34.5)	0.105
**Gamma-glutamyltransferase ≥ 48 U/L (%)**	263 (45.4)	183 (41.3)	0.083	223 (42.4)	71 (30.2)	0.255
**Alkaline phosphatase ≥ 140 U/L (%)**	257 (44.4)	225 (50.8)	0.128	236 (44.9)	104 (44.3)	0.012
**Platelet ≤ 150 10^9/L (%)**	67 (11.6)	45 (10.2)	0.045	58 (11.0)	24 (10.2)	0.026
**Creatinine > 120 μmol/L (%)**	94 (16.2)	66 (14.9)	0.037	76 (14.4)	25 (10.6)	0.115

Blood test measurements are indicated as the presence or absence of a particular measurement above or below the stated threshold.

Additional file 4. Subgroup analyses showing Kaplan-Meier plots of the propensity-matched cohort for all-cause mortality. Flow diagram depicts analysis design. P values from logrank tests were indicated. Hazard ratios from Cox proportional hazards regression analyses were indicated. Numbers in parentheses indicate the 95% confidence intervals.

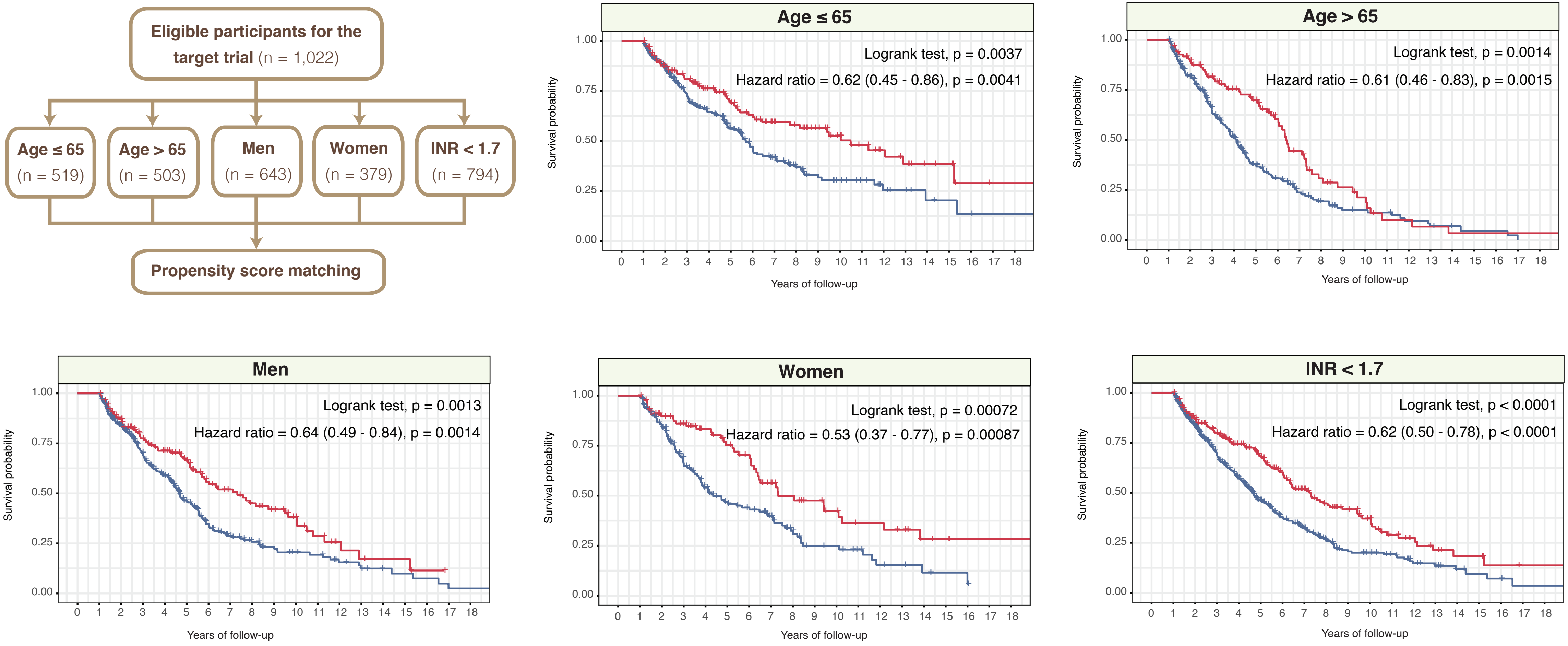

Additional file 5: Baseline characteristics of the unmatched and matched cohorts in individuals aged ≤ 65

	Unmatched cohort			Matched cohort		
**Characteristics**	**No warfarin**	**Warfarin**	**Standardised difference**	**No warfarin**	**Warfarin**	**Standardised difference**
**N**	263	256		239	136	
**Gender, women (%)**	65 (24.7)	86 (33.6)	0.196	63 (26.4)	44 (32.4)	0.132
**Age at baseline, years (mean (SD))**	54.64 (8.03)	52.35 (8.71)	0.273	54.25 (8.08)	52.04 (8.83)	0.262
**Type 2 diabetes (%)**	48 (18.3)	47 (18.4)	0.003	43 (18.0)	24 (17.6)	0.009
**Hypertension (%)**	87 (33.1)	71 (27.7)	0.116	78 (32.6)	35 (25.7)	0.152
**Ascites (%)**	76 (28.9)	90 (35.2)	0.134	64 (26.8)	45 (33.1)	0.138
**Hepatic encephalopathy (%)**	19 (7.2)	26 (10.2)	0.104	17 (7.1)	10 (7.4)	0.009
**Renal disease (%)**	37 (14.1)	31 (12.1)	0.058	26 (10.9)	10 (7.4)	0.123
**Steatosis (%)**	36 (13.7)	32 (12.5)	0.035	32 (13.4)	19 (14.0)	0.017
**Lipid regulating drugs (%)**	59 (22.4)	57 (22.3)	0.004	56 (23.4)	30 (22.1)	0.033
**Hypertension treatment (%)**	87 (33.1)	85 (33.2)	0.003	80 (33.5)	42 (30.9)	0.055
**Digoxin (%)**	37 (14.1)	22 (8.6)	0.173	28 (11.7)	10 (7.4)	0.149
**Varices (%)**	46 (17.5)	46 (18.0)	0.013	36 (15.1)	21 (15.4)	0.011
**Bilirubin ≥ 34.2 μmol/L (%)**	77 (29.3)	61 (23.8)	0.124	62 (25.9)	29 (21.3)	0.109
**Albumin < 35 g/L (%)**	10 (3.8)	15 (5.9)	0.096	9 (3.8)	9 (6.6)	0.129
**INR ≥ 1.7 (%)**	7 (2.7)	123 (48.0)	1.223	7 (2.9)	8 (5.9)	0.144
**Alanine aminotransferase ≥ 35 U/L (%)**	125 (47.5)	103 (40.2)	0.147	108 (45.2)	51 (37.5)	0.157
**Aspartate transaminase ≥ 40 U/L (%)**	65 (24.7)	58 (22.7)	0.048	58 (24.3)	25 (18.4)	0.144
**BMI ≥ 30 kg/m2 (%)**	94 (35.7)	83 (32.4)	0.07	81 (33.9)	39 (28.7)	0.113
**Gamma-glutamyltransferase ≥ 48 U/L (%)**	142 (54.0)	100 (39.1)	0.303	119 (49.8)	40 (29.4)	0.426
**Alkaline phosphatase ≥ 140 U/L (%)**	116 (44.1)	126 (49.2)	0.103	104 (43.5)	57 (41.9)	0.032
**Platelet ≤ 150 10^9/L (%)**	28 (10.6)	28 (10.9)	0.009	24 (10.0)	13 (9.6)	0.016
**Creatinine > 120 μmol/L (%)**	24 (9.1)	27 (10.5)	0.048	18 (7.5)	10 (7.4)	0.007

Blood test measurements are indicated as the presence or absence of a particular measurement above or below the stated threshold.

Additional file 6: Baseline characteristics of the unmatched and matched cohorts in individuals aged > 65

	Unmatched cohort			Matched cohort		
Characteristics	No warfarin	Warfarin	Standardised difference	No warfarin	Warfarin	Standardised difference
N	316	187		266	97	
Gender, women (%)	134 (42.4)	94 (50.3)	0.158	117 (44.0)	45 (46.4)	0.048
Age at baseline, years (mean (SD))	74.83 (6.89)	74.47 (6.33)	0.055	74.45 (6.82)	74.26 (6.32)	0.030
Type 2 diabetes (%)	88 (27.8)	52 (27.8)	0.001	67 (25.2)	23 (23.7)	0.034
Hypertension (%)	171 (54.1)	112 (59.9)	0.117	134 (50.4)	43 (44.3)	0.121
Ascites (%)	67 (21.2)	35 (18.7)	0.062	55 (20.7)	21 (21.6)	0.024
Hepatic encephalopathy (%)	10 (3.2)	6 (3.2)	0.003	9 (3.4)	4 (4.1)	0.039
Renal disease (%)	41 (13.0)	27 (14.4)	0.043	29 (10.9)	12 (12.4)	0.046
Steatosis (%)	27 (8.5)	16 (8.6)	<0.001	24 (9.0)	8 (8.2)	0.028
Lipid regulating drugs (%)	101 (32.0)	73 (39.0)	0.148	82 (30.8)	31 (32.0)	0.024
Hypertension treatment (%)	149 (47.2)	109 (58.3)	0.224	127 (47.7)	52 (53.6)	0.117
Digoxin (%)	70 (22.2)	43 (23.0)	0.020	47 (17.7)	16 (16.5)	0.031
Varices (%)	49 (15.5)	28 (15.0)	0.015	40 (15.0)	14 (14.4)	0.017
Bilirubin ≥ 34.2 μmol/L (%)	53 (16.8)	32 (17.1)	0.009	44 (16.5)	12 (12.4)	0.119
Albumin < 35 g/L (%)	8 (2.5)	6 (3.2)	0.041	7 (2.6)	4 (4.1)	0.083
INR ≥ 1.7 (%)	4 (1.3)	94 (50.3)	1.353	4 (1.5)	4 (4.1)	0.159
Alanine aminotransferase ≥ 35 U/L (%)	121 (38.3)	76 (40.6)	0.048	91 (34.2)	31 (32.0)	0.048
Aspartate transaminase ≥ 40 U/L (%)	54 (17.1)	30 (16.0)	0.028	45 (16.9)	13 (13.4)	0.098
BMI ≥ 30 kg/m2 (%)	133 (42.1)	86 (46.0)	0.08	116 (43.6)	43 (44.3)	0.015
Gamma-glutamyltransferase ≥ 48 U/L (%)	121 (38.3)	83 (44.4)	0.124	94 (35.3)	31 (32.0)	0.072
Alkaline phosphatase ≥ 140 U/L (%)	141 (44.6)	99 (52.9)	0.167	117 (44.0)	43 (44.3)	0.007
Platelet ≤ 150 10^9/L (%)	39 (12.3)	17 (9.1)	0.105	31 (11.7)	10 (10.3)	0.043
Creatinine > 120 μmol/L (%)	70 (22.2)	39 (20.9)	0.032	45 (16.9)	14 (14.4)	0.068

Blood test measurements are indicated as the presence or absence of a particular measurement above or below the stated threshold.

Additional file 7: Baseline characteristics of the unmatched and matched cohorts in men

	Unmatched cohort			Matched cohort		
Characteristics	No warfarin	Warfarin	Standardised difference	No warfarin	Warfarin	Standardised difference
N	380	263		333	142	
Age at baseline, years (mean (SD))	63.63 (12.36)	60.04 (12.44)	0.290	62.82 (12.62)	59.53 (13.14)	0.255
Type 2 diabetes (%)	93 (24.5)	58 (22.1)	0.057	75 (22.5)	26 (18.3)	0.105
Hypertension (%)	155 (40.8)	106 (40.3)	0.010	130 (39.0)	49 (34.5)	0.094
Ascites (%)	102 (26.8)	80 (30.4)	0.079	92 (27.6)	45 (31.7)	0.089
Hepatic encephalopathy (%)	23 (6.1)	22 (8.4)	0.089	19 (5.7)	11 (7.7)	0.082
Renal disease (%)	50 (13.2)	34 (12.9)	0.007	42 (12.6)	15 (10.6)	0.064
Steatosis (%)	36 (9.5)	26 (9.9)	0.014	33 (9.9)	12 (8.5)	0.051
Lipid regulating drugs (%)	95 (25.0)	76 (28.9)	0.088	85 (25.5)	33 (23.2)	0.053
Hypertension treatment (%)	155 (40.8)	121 (46.0)	0.105	140 (42.0)	61 (43.0)	0.019
Digoxin (%)	66 (17.4)	37 (14.1)	0.091	55 (16.5)	19 (13.4)	0.088
Varices (%)	68 (17.9)	44 (16.7)	0.031	45 (13.5)	18 (12.7)	0.025
Bilirubin ≥ 34.2 µmol/L (%)	89 (23.4)	55 (20.9)	0.060	71 (21.3)	31 (21.8)	0.012
Albumin < 35 g/L (%)	13 (3.4)	15 (5.7)	0.110	11 (3.3)	8 (5.6)	0.113
INR ≥ 1.7 (%)	8 (2.1)	128 (48.7)	1.266	8 (2.4)	9 (6.3)	0.193
Alanine aminotransferase ≥ 35 U/L (%)	159 (41.8)	102 (38.8)	0.062	135 (40.5)	50 (35.2)	0.110
Aspartate transaminase ≥ 40 U/L (%)	78 (20.5)	53 (20.2)	0.009	68 (20.4)	24 (16.9)	0.090
BMI ≥ 30 kg/m2 (%)	142 (37.4)	102 (38.8)	0.03	117 (35.1)	48 (33.8)	0.028
Gamma-glutamyltransferase ≥ 48 U/L (%)	188 (49.5)	110 (41.8)	0.154	152 (45.6)	47 (33.1)	0.259
Alkaline phosphatase ≥ 140 U/L (%)	150 (39.5)	123 (46.8)	0.148	135 (40.5)	62 (43.7)	0.063
Platelet ≤ 150 10^9/L (%)	47 (12.4)	25 (9.5)	0.092	35 (10.5)	14 (9.9)	0.022
Creatinine > 120 µmol/L (%)	58 (15.3)	48 (18.3)	0.080	49 (14.7)	20 (14.1)	0.018

Blood test measurements are indicated as the presence or absence of a particular measurement above or below the stated threshold.

Additional file 8: Baseline characteristics of the unmatched and matched cohorts in women

	Unmatched cohort			Matched cohort		
Characteristics	No warfarin	Warfarin	Standardised difference	No warfarin	Warfarin	Standardised difference
N	199	180		164	90	
Age at baseline, years (mean (SD))	69.54 (11.86)	64.10 (14.44)	0.412	68.85 (11.80)	63.40 (14.65)	0.409
Type 2 diabetes (%)	43 (21.6)	41 (22.8)	0.028	34 (20.7)	18 (20.0)	0.018
Hypertension (%)	103 (51.8)	77 (42.8)	0.181	75 (45.7)	28 (31.1)	0.304
Ascites (%)	41 (20.6)	45 (25.0)	0.105	34 (20.7)	21 (23.3)	0.063
Hepatic encephalopathy (%)	6 (3.0)	10 (5.6)	0.126	6 (3.7)	5 (5.6)	0.091
Renal disease (%)	28 (14.1)	24 (13.3)	0.021	18 (11.0)	7 (7.8)	0.110
Steatosis (%)	27 (13.6)	22 (12.2)	0.040	24 (14.6)	13 (14.4)	0.005
Lipid regulating drugs (%)	65 (32.7)	54 (30.0)	0.057	54 (32.9)	27 (30.0)	0.063
Hypertension treatment (%)	81 (40.7)	73 (40.6)	0.003	64 (39.0)	31 (34.4)	0.095
Digoxin (%)	41 (20.6)	28 (15.6)	0.131	28 (17.1)	9 (10.0)	0.208
Varices (%)	27 (13.6)	30 (16.7)	0.087	24 (14.6)	14 (15.6)	0.026
Bilirubin ≥ 34.2 μmol/L (%)	41 (20.6)	38 (21.1)	0.013	30 (18.3)	12 (13.3)	0.136
Albumin < 35 g/L (%)	5 (2.5)	6 (3.3)	0.049	4 (2.4)	4 (4.4)	0.110
INR ≥ 1.7 (%)	3 (1.5)	89 (49.4)	1.317	3 (1.8)	3 (3.3)	0.095
Alanine aminotransferase ≥ 35 U/L (%)	87 (43.7)	77 (42.8)	0.019	68 (41.5)	34 (37.8)	0.075
Aspartate transaminase ≥ 40 U/L (%)	41 (20.6)	35 (19.4)	0.029	29 (17.7)	14 (15.6)	0.057
BMI ≥ 30 kg/m2 (%)	85 (42.7)	67 (37.2)	0.11	69 (42.1)	31 (34.4)	0.157
Gamma-glutamyltransferase ≥ 48 U/L (%)	75 (37.7)	73 (40.6)	0.059	57 (34.8)	24 (26.7)	0.176
Alkaline phosphatase ≥ 140 U/L (%)	107 (53.8)	102 (56.7)	0.058	80 (48.8)	38 (42.2)	0.132
Platelet ≤ 150 10^9/L (%)	20 (10.1)	20 (11.1)	0.034	18 (11.0)	10 (11.1)	0.004
Creatinine > 120 μmol/L (%)	36 (18.1)	18 (10.0)	0.234	13 (7.9)	4 (4.4)	0.145

Blood test measurements are indicated as the presence or absence of a particular measurement above or below the stated threshold.

Additional file 9: Baseline characteristics of the unmatched and matched cohorts in individuals with INR < 1.7

	Unmatched cohort			Matched cohort		
**Characteristics**	**No warfarin**	**Warfarin**	**Standardised difference**	**No warfarin**	**Warfarin**	**Standardised difference**
**N**	568	226		471	217	
**Gender, women (%)**	196 (34.5)	91 (40.3)	0.119	173 (36.7)	85 (39.2)	0.050
**Age at baseline, years (mean (SD))**	65.74 (12.47)	61.20 (13.62)	0.348	64.64 (12.43)	61.90 (13.33)	0.213
**Type 2 diabetes (%)**	133 (23.4)	45 (19.9)	0.085	111 (23.6)	43 (19.8)	0.091
**Hypertension (%)**	251 (44.2)	76 (33.6)	0.218	198 (42.0)	76 (35.0)	0.145
**Ascites (%)**	137 (24.1)	63 (27.9)	0.086	117 (24.8)	60 (27.6)	0.064
**Hepatic encephalopathy (%)**	26 (4.6)	15 (6.6)	0.090	24 (5.1)	12 (5.5)	0.019
**Renal disease (%)**	74 (13.0)	20 (8.8)	0.134	54 (11.5)	19 (8.8)	0.090
**Steatosis (%)**	62 (10.9)	25 (11.1)	0.005	57 (12.1)	24 (11.1)	0.033
**Lipid regulating drugs (%)**	156 (27.5)	58 (25.7)	0.041	128 (27.2)	57 (26.3)	0.021
**Hypertension treatment (%)**	232 (40.8)	88 (38.9)	0.039	196 (41.6)	84 (38.7)	0.059
**Digoxin (%)**	104 (18.3)	26 (11.5)	0.192	76 (16.1)	25 (11.5)	0.134
**Varices (%)**	89 (15.7)	32 (14.2)	0.042	66 (14.0)	32 (14.7)	0.021
**Bilirubin ≥ 34.2 μmol/L (%)**	124 (21.8)	41 (18.1)	0.092	93 (19.7)	39 (18.0)	0.045
**Albumin < 35 g/L (%)**	17 (3.0)	11 (4.9)	0.097	17 (3.6)	10 (4.6)	0.050
**Alanine aminotransferase ≥ 35 U/L (%)**	240 (42.3)	78 (34.5)	0.160	187 (39.7)	76 (35.0)	0.097
**Aspartate transaminase ≥ 40 U/L (%)**	115 (20.2)	39 (17.3)	0.077	90 (19.1)	37 (17.1)	0.053
**BMI ≥ 30 kg/m2 (%)**	223 (39.3)	78 (34.5)	0.10	180 (38.2)	76 (35.0)	0.066
**Gamma-glutamyltransferase ≥ 48 U/L (%)**	258 (45.4)	67 (29.6)	0.330	186 (39.5)	67 (30.9)	0.181
**Alkaline phosphatase ≥ 140 U/L (%)**	251 (44.2)	98 (43.4)	0.017	204 (43.3)	93 (42.9)	0.009
**Platelet ≤ 150 10^9/L (%)**	64 (11.3)	24 (10.6)	0.021	54 (11.5)	22 (10.1)	0.043
**Creatinine > 120 μmol/L (%)**	90 (15.8)	22 (9.7)	0.184	72 (15.3)	22 (10.1)	0.155

Blood test measurements are indicated as the presence or absence of a particular measurement above or below the stated threshold.

Additional file 10. Summary of survey responses.

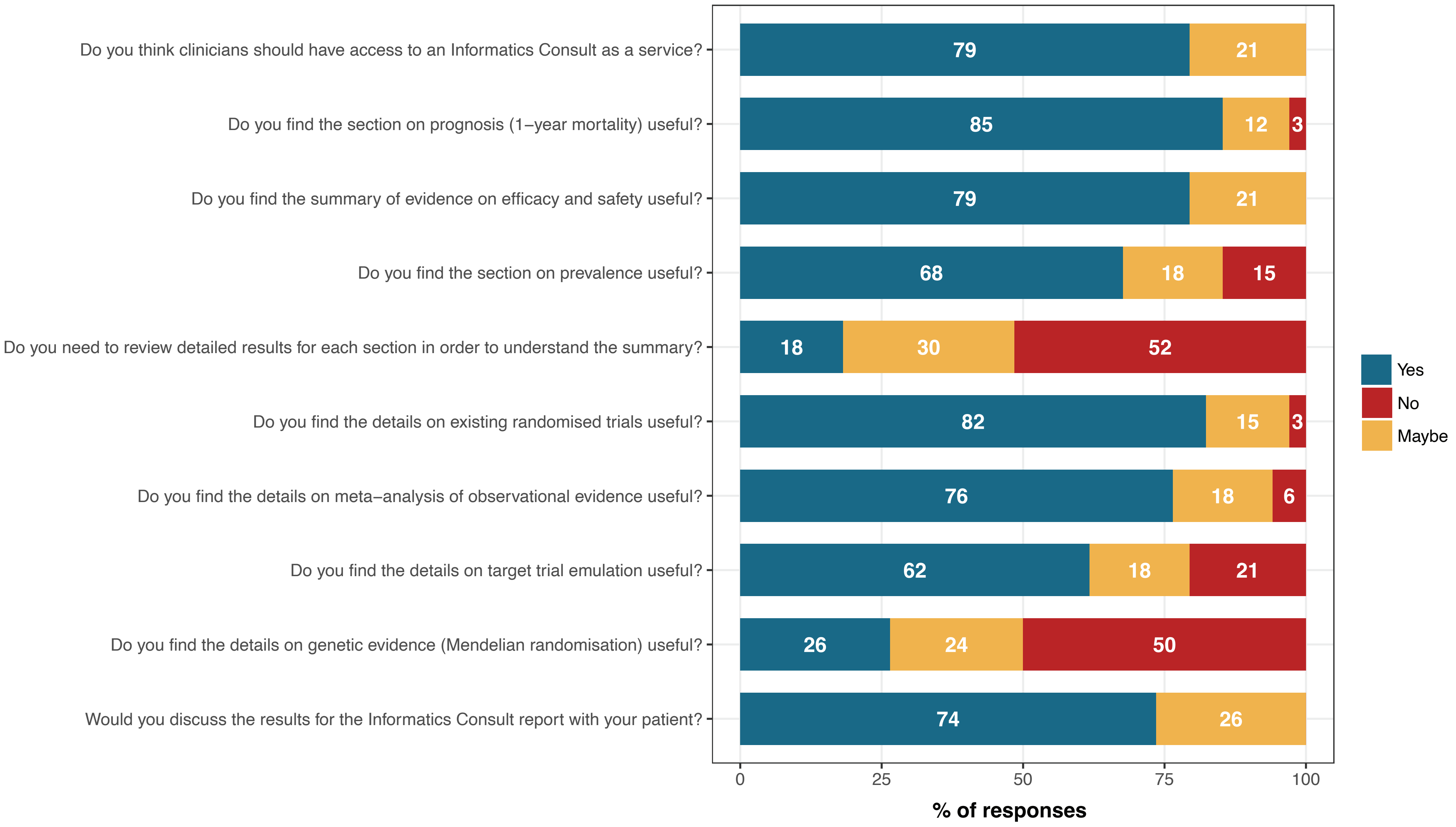

Additional file 11. Survey questionnaire.

1. Do you think clinicians should have access to an Informatics Consult as a service?
   Yes | No | Maybe

2. Do you find the section on prognosis (1−year mortality) useful?
   Yes | No | Maybe

3. Do you find the summary of evidence on efficacy and safety useful?
   Yes | No | Maybe

4. Do you find the section on prevalence useful?
   Yes | No | Maybe

5. Do you need to review detailed results for each section in order to understand the summary?
   Yes | No | Maybe

6. Do you find the details on existing randomised trials useful?
   Yes | No | Maybe

7. Do you find the details on meta−analysis of observational evidence useful?
   Yes | No | Maybe

8. Do you find the details on target trial emulation useful?
   Yes | No | Maybe

9. Do you find the details on genetic evidence (Mendelian randomisation) useful?
   Yes | No | Maybe

10. Would you discuss the results for the Informatics Consult report with your patient?
    Yes | No | Maybe
